# Supplementary material for: Ancestral functionality and symbiotic refinement of NIN in root nodule symbiosis
Source: Nat Commun. 2026 Apr 6;17:4907. doi: 10.1038/s41467-026-71330-1 (PMC13230612; doi:10.1038/s41467-026-71330-1)
Supplement: Supplementary file 1 — Supplementary Information [file 41467_2026_71330_MOESM1_ESM.pdf]

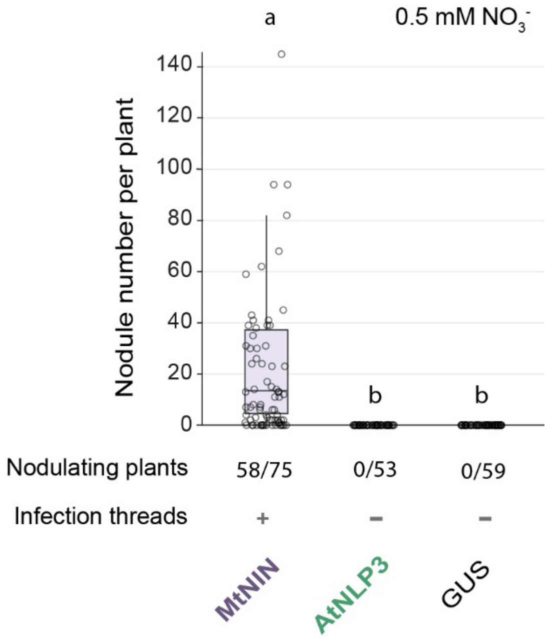

Supplementary Fig. 1 | AtNLP3 is not functional in root nodule symbiosis. Number of nodules formed on *Mtnin-1* mutant roots complemented with MtNIN and AtNLP3. Plants were harvested at 4 weeks post inoculation with *S. meliloti* 2011 expressing GFP. Box plots show the median (centre line), interquartile range (box), and whiskers extending to the most extreme values within 1.5× the interquartile range of the number of nodules per nodulating plant; points represent individual observations. Lowercase letters indicate significant differences between samples (Kruskal-Wallis and post-hoc Dunn's test, Benjamini-Yekutieli adjusted $p < 0.05$). Source data are provided as a Source Data file.

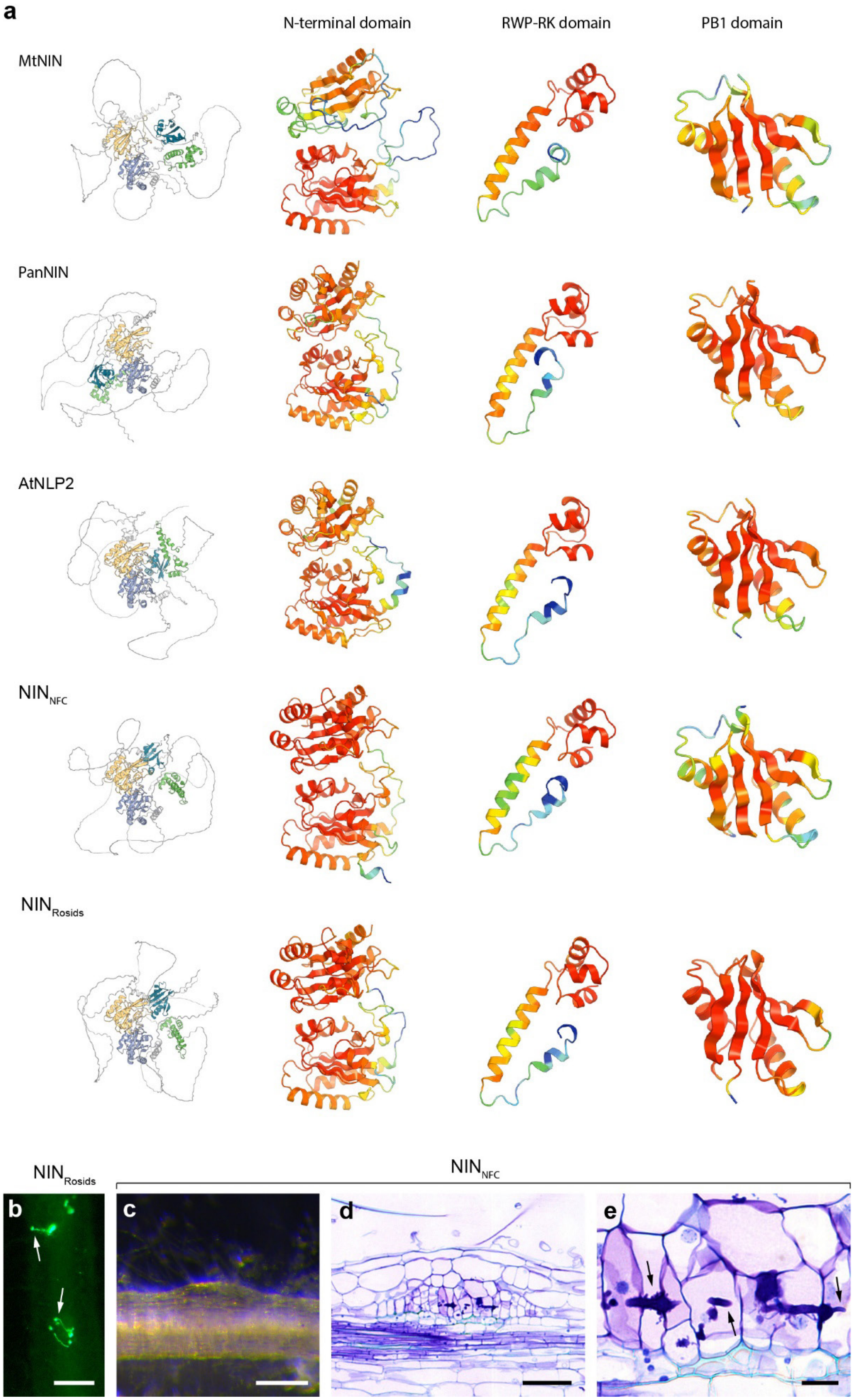

a
N-terminal domain
RWP-RK domain
PB1 domain
MtNIN
PanNIN
AtNLP2
NIN$_{NFC}$
NIN$_{Rosids}$
NIN$_{Rosids}$
NIN$_{NFC}$
b
c
d
e

Supplementary Fig. 2 | Functionality of resurrected ancestral NIN proteins. a, Predicted models of extant NINs and resurrected ancestors share structural conserved domains. Overview of full-length NIN and three conserved domains; N-terminal domain, RWP-RK DNA-binding domain, and PB1 protein-protein interaction domain, colored by their pLDDT score, with red indicating a score of 90 and higher. **b**, Green fluorescence stereomicroscopy images showing infection threads (arrows) formed on *Mtnin-1* roots complemented with the resurrected NIN_{Rosids} ancestor. Scale bars: 2 mm. **c**, Stereomicroscope images showing a nodule primordium formed on *Mtnin-1* roots transformed with the NIN_{NFC} ancestor. Scale bar: 2 mm. **d**, Longitudinal section of NIN_{NFC} induced nodule primordium, stained with toluidine blue. Scale bar: 100 µm. **e**, Magnification of (**d**). Arrows indicate infection threads. Scale bar: 20 µm.

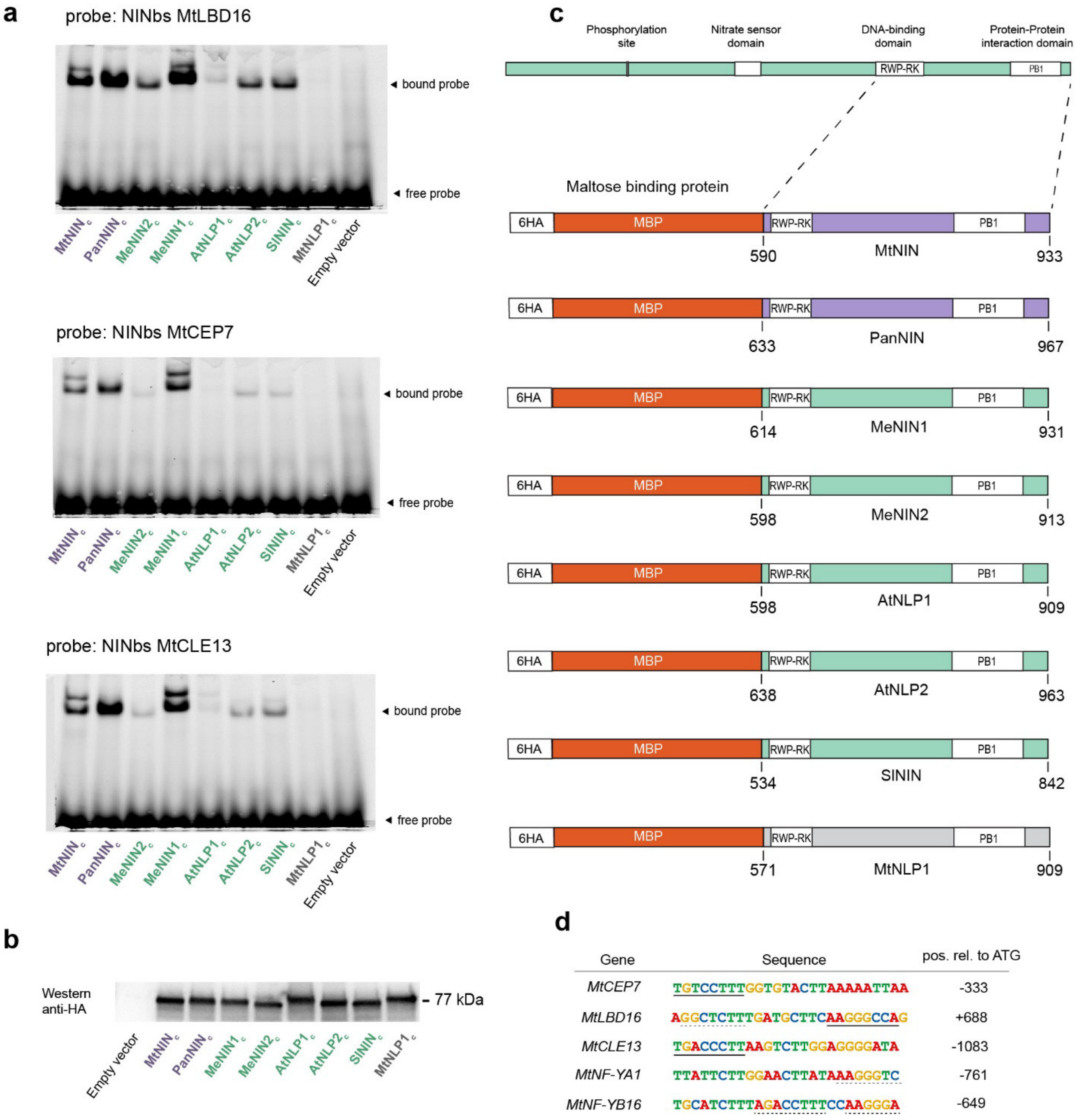

Supplementary Fig. 3 | C-terminus of different NIN-orthologs can bind to symbiotic NIN-binding sites *in vitro*. a, Electrophoretic mobility shift assay (EMSA) testing the binding of the C-terminus of different NIN-orthologs, fused with 6xHAtag-MBP, to fluorescently labelled NIN *cis*-regulatory binding sites (NINbs) of *MtLBD16, MtCEP7,* and *MtCLE13*. **b**, Western blot showing the relative concentration of different fusion proteins used in EMSA. An uncropped image is provided in the source data file. **c**, Constructs used in the EMSA (Fig. 2a, Supplementary Fig. 3a). The C-terminal region of different NIN orthologs was used, which includes the DNA-

binding domain (RWP-RK) and a protein-protein interaction domain (PB1). Numbers refer to the amino acid range of each NIN protein that was used. The NIN C-terminal region was fused to the maltose binding protein (MBP) to aid in solubility, and 6 repetitions of the hemagglutinin tag (6HA) for detection. **d**, Probe sequences that were used in EMSA (Fig. 2a, Supplementary Fig. 3a). Solid lines indicate full match to the AtNLP2 binding site described by [18], dashed lines indicate partial match. The position relative to the ATG start codon is indicated. Note that the NIN binding site of MtLBD16 is in the first intron [27].

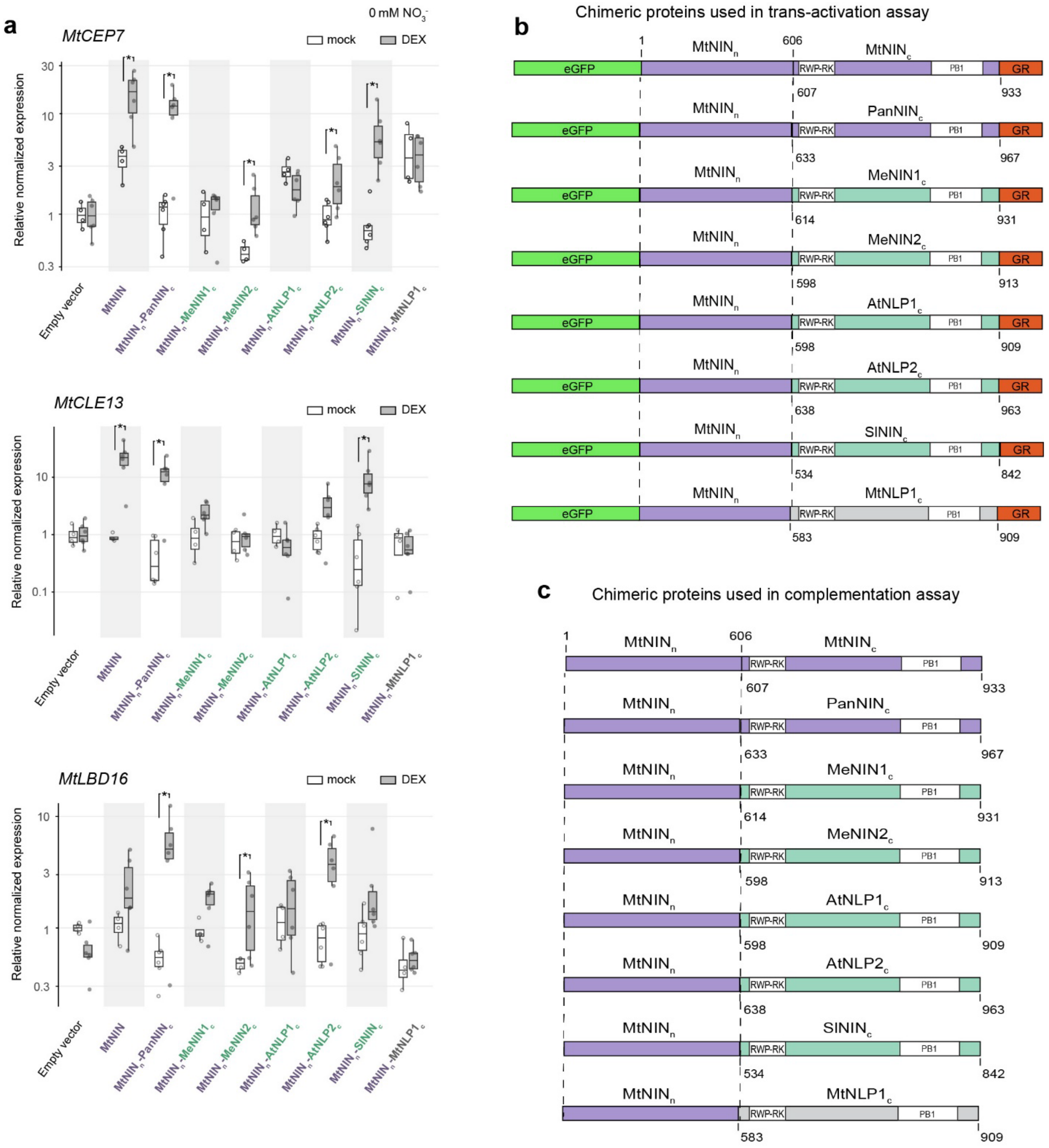

Supplementary Fig. 4 | Symbiotic functionality of chimeric NIN proteins. a, qRT-PCR showing chimeric NIN proteins induce *MtCEP7*, *MtCLE13* and *MtLBD16* expression in a transactivation assay. Medicago roots producing the N-terminus of MtNIN fused to the C-terminus of different NIN/NLPs and the rat glucocorticoid receptor (Supplementary Fig. 4b), were treated with 10 µM dexamethasone (DEX) or DMSO (mock) for 16 hours. Four (mock) and six (dex)

independent biological replicates were used for protein. Expression levels were normalized to the average expression of mock treated empty vector roots. Asterisks indicate significant differences (Mann-Whitney U-test, 2-sided, $p < 0.05$). Source data are provided as a Source Data file. **b**, Chimeric NIN proteins used in the transactivation assay (Fig. 2b, Supplementary Fig. 4a). The N-terminal region (residue 1-606) of MtNIN was fused to the C-terminal region of different NIN orthologs, which includes the RWP-RK domain and the PB1 domain. Numbers below each C-terminus show the amino acid range of each protein that was used. GR indicates the rat glucocorticoid receptor. **c**, Chimeric NIN proteins used in the complementation assay (Fig. 2c,d). The N-terminal region (residues 1-606) of MtNIN was fused to the C-terminal region of different NIN-orthologs, which includes the RWP-RK domain and the PB1 domain. Numbers below each C-terminus show the amino acid range of each protein that was used.

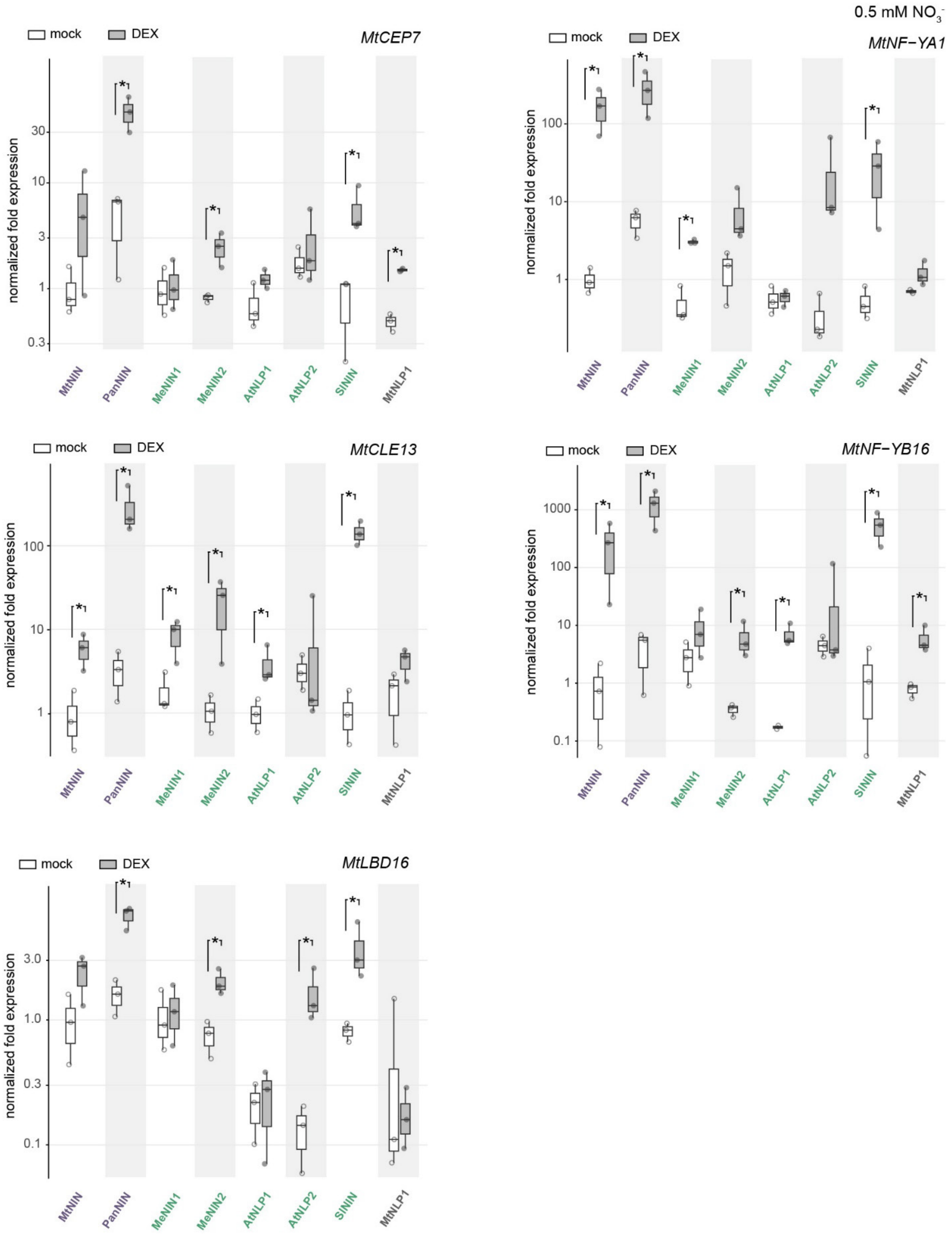

0.5 mM NO_3^-
mock
DEX
MtCEP7
normalized fold expression
30
10
3
1
0.3
MtNF−YA1
100
10
1
MtCLE13
100
10
1
MtNF−YB16
1000
100
10
1
0.1
MtLBD16
3.0
1.0
0.3
0.1
*
MtNIN
PanNIN
MeNIN1
MeNIN2
AtNLP1
AtNLP2
SlNIN
MtNLP1

Supplementary Fig. 5 | Transactivation assay using non-chimeric NIN-GR fusions. qRT-PCR showing induction of NIN target genes by non-chimeric NIN proteins fused to GR. Medicago roots expressing the constructs, were treated with 10 µM dexamethasone (DEX) or DMSO (mock) for 16 hours. Three independent biological replicates were analyzed per treatment. Expression levels were normalized to the average expression of mock-treated roots expressing MtNIN-GR. Asterisks indicate significant differences (Student's t-test on log-transformed data, 2-sided, $p < 0.05$). Source data are provided as a Source Data file.

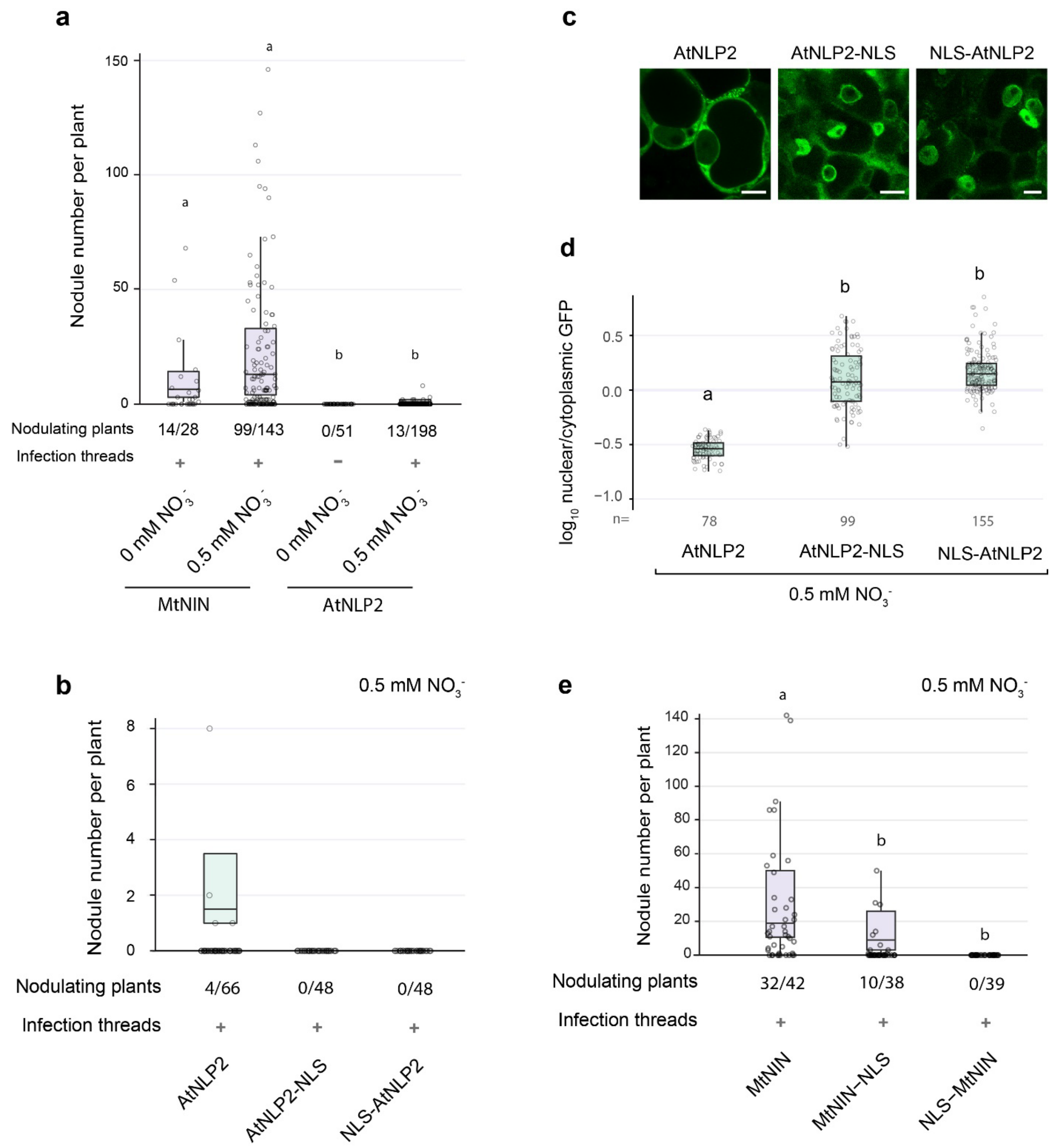

Supplementary Fig. 6 | MtNIN, but not AtNLP2, can function in nodulation in absence of nitrate. a, Number of nodules formed on *Mtnin-1* mutant roots complemented with MtNIN or AtNLP2 under different exogenous nitrate concentrations. Plants were harvested at 4 weeks post inoculation with *S. meliloti* 2011 expressing GFP. Box plots show the number of nodules per nodulated plant. Lowercase letters indicate significant differences between samples (Kruskal-Wallis and post-hoc Dunn's test, Benjamini-Yekutieli adjusted $p < 0.05$). **b**, Number of nodules

formed on *Mtnin-1* mutant roots complemented with *AtNLP2* or *AtNLP2* fused with nuclear localization signals (NLS) at 0.5 mM nitrate. Differences are not significant (Kruskal-Wallis and post-hoc Dunn's test, Benjamini-Yekutieli adjusted $p < 0.05$). **c**, Confocal images showing the subcellular localization of GFP-tagged AtNLP2 or AtNLP2 fused with nuclear localization signals (NLS) at 0.5 mM nitrate. Scale bars: 10 µm. **d**, Quantification of subcellular localization in (**c**). Lowercase letters indicate significant differences between samples (Kruskal-Wallis and post-hoc Dunn's test, Benjamini-Yekutieli adjusted, $p < 0.05$). N indicates the number of cells analyzed. **e**, Number of nodules formed on *Mtnin-1* mutant roots complemented with MtNIN or MtNIN fused with nuclear localization signals (NLS) at 0.5 mM nitrate. Lowercase letters indicate significant differences between samples (Kruskal-Wallis and post-hoc Dunn's test, Benjamini-Yekutieli adjusted $p < 0.05$). Source data of panels a-c are provided as a Source Data file. All box plots show the median (centre line), interquartile range (box), and whiskers extending to the most extreme values within 1.5× the interquartile range; points represent individual observations.

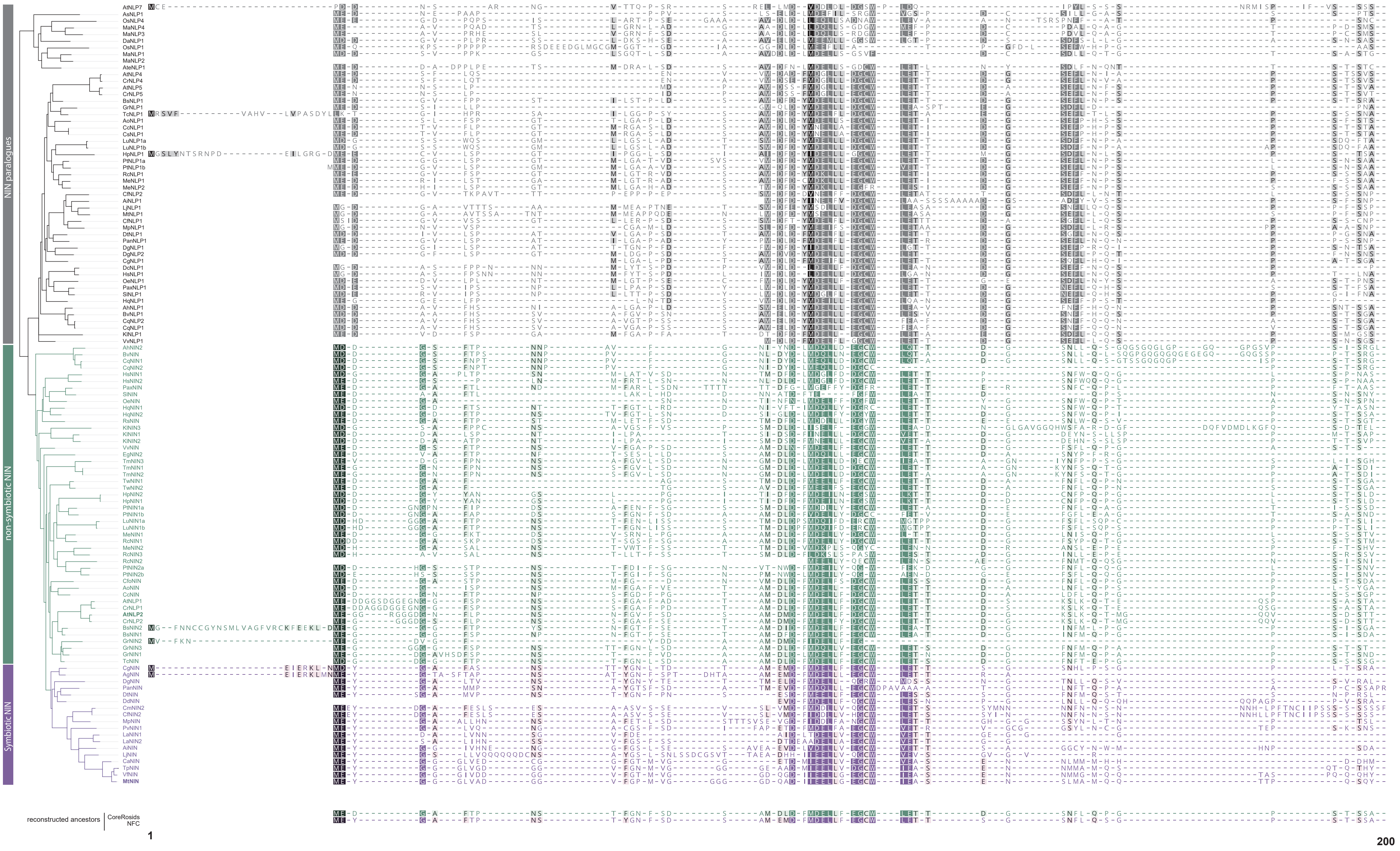
NIN paralogues
non-symbiotic NIN
Symbiotic NIN
reconstructed ancestors
CoreRosids
NFC
1
200

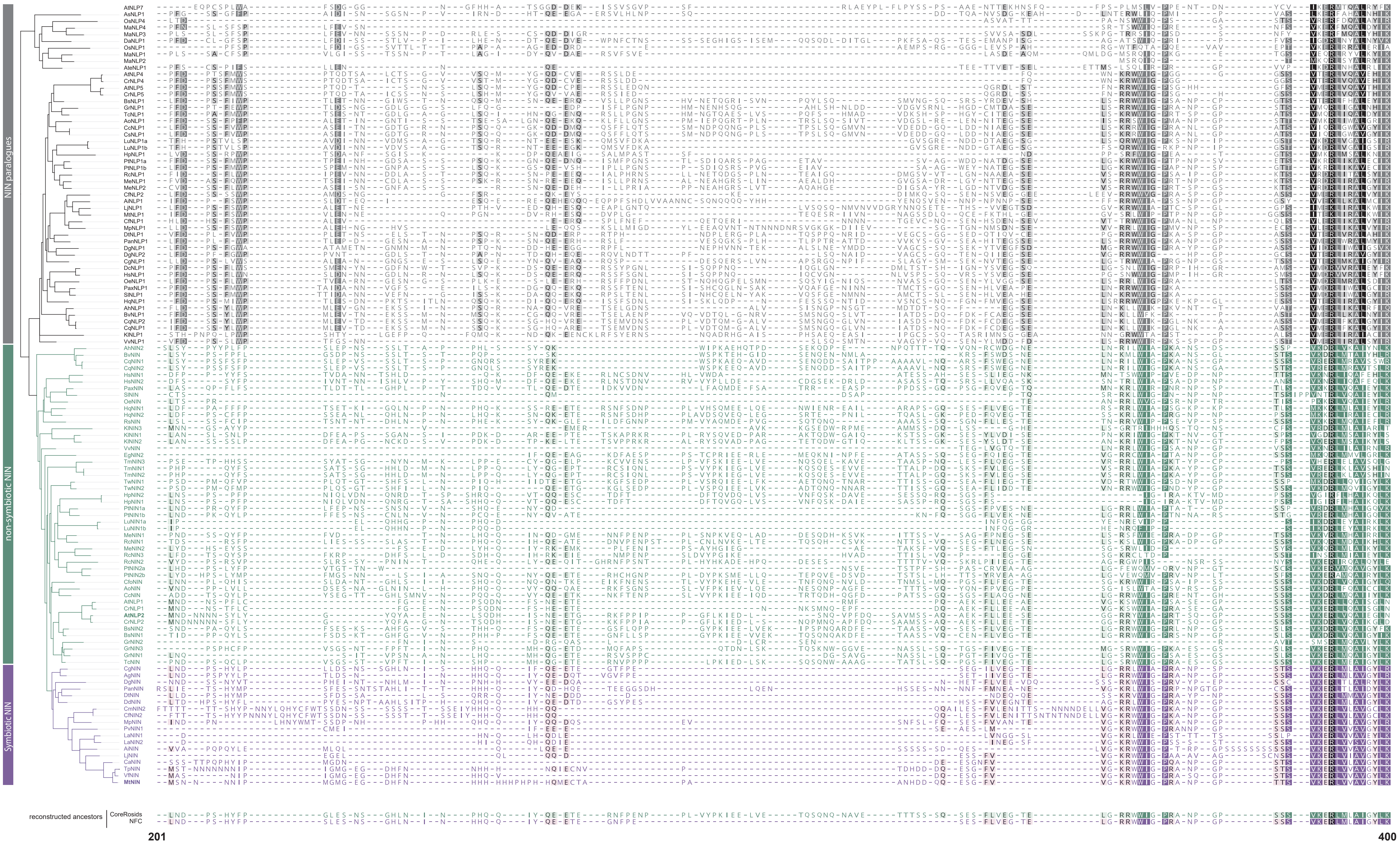
NIN paralogues
non-symbiotic NIN
Symbiotic NIN
reconstructed ancestors
CoreRosids
NFC
201
400

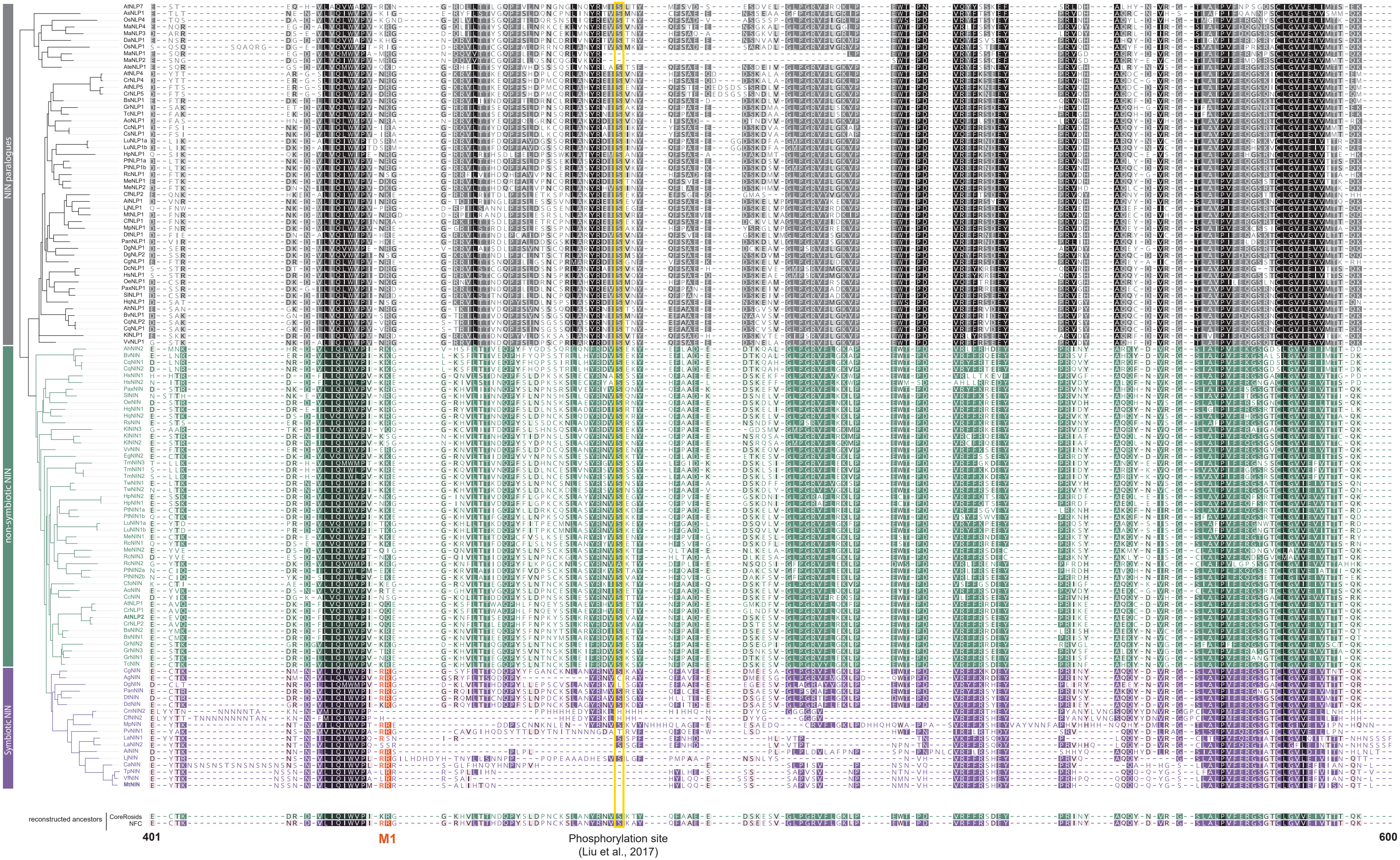

NIN paralogues
non-symbiotic NIN
Symbiotic NIN
reconstructed ancestors
CoreRosids
NFC
401
M1
Phosphorylation site
(Liu et al., 2017)
600

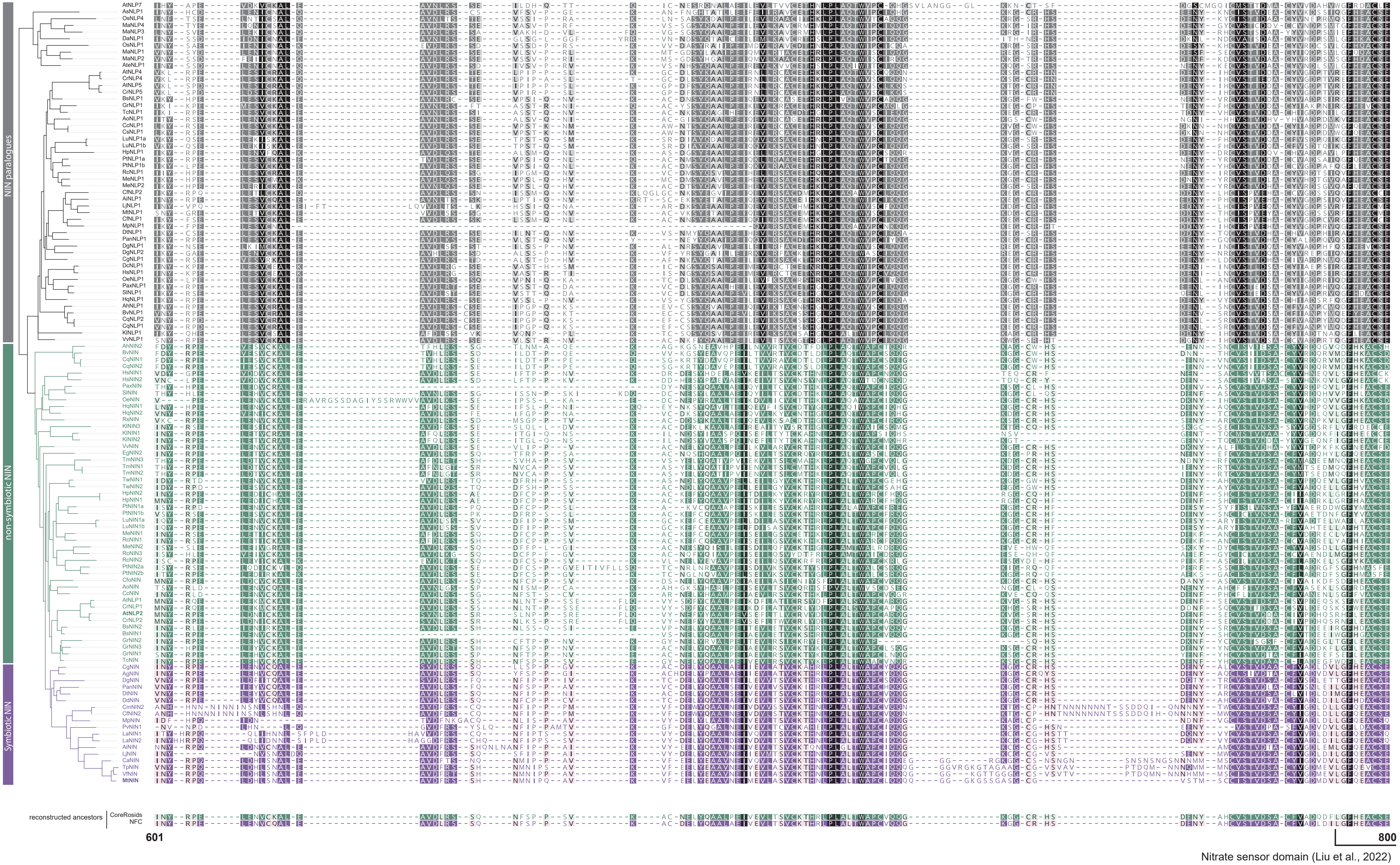
NIN paralogues
non-symbiotic NIN
Symbiotic NIN
reconstructed ancestors
CoreRosids
NFC
601
800
Nitrate sensor domain (Liu et al., 2022)

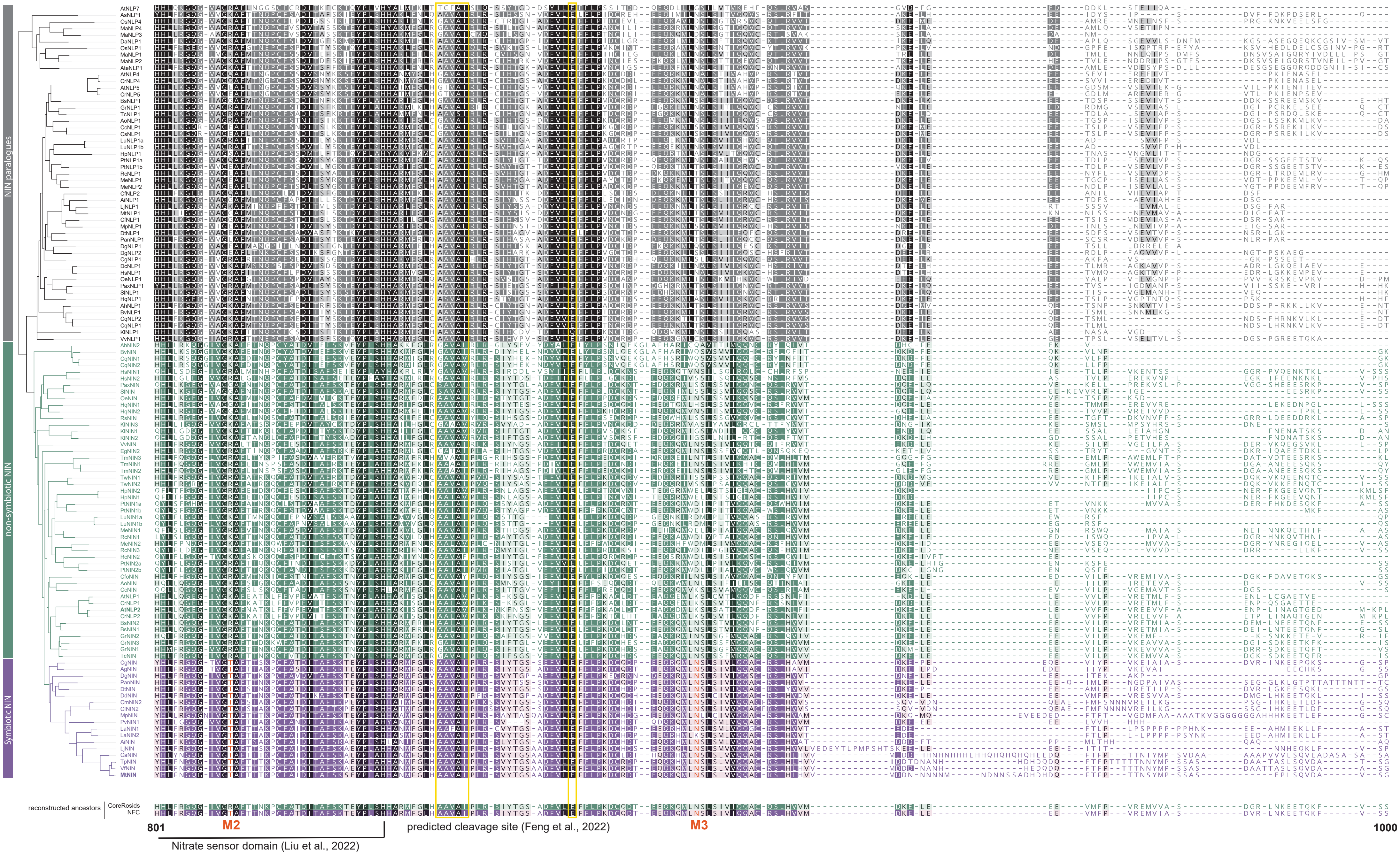
NIN paralogues
non-symbiotic NIN
Symbiotic NIN
reconstructed ancestors
CoreRosids
NFC
801
M2
predicted cleavage site (Feng et al., 2022)
M3
1000
Nitrate sensor domain (Liu et al., 2022)

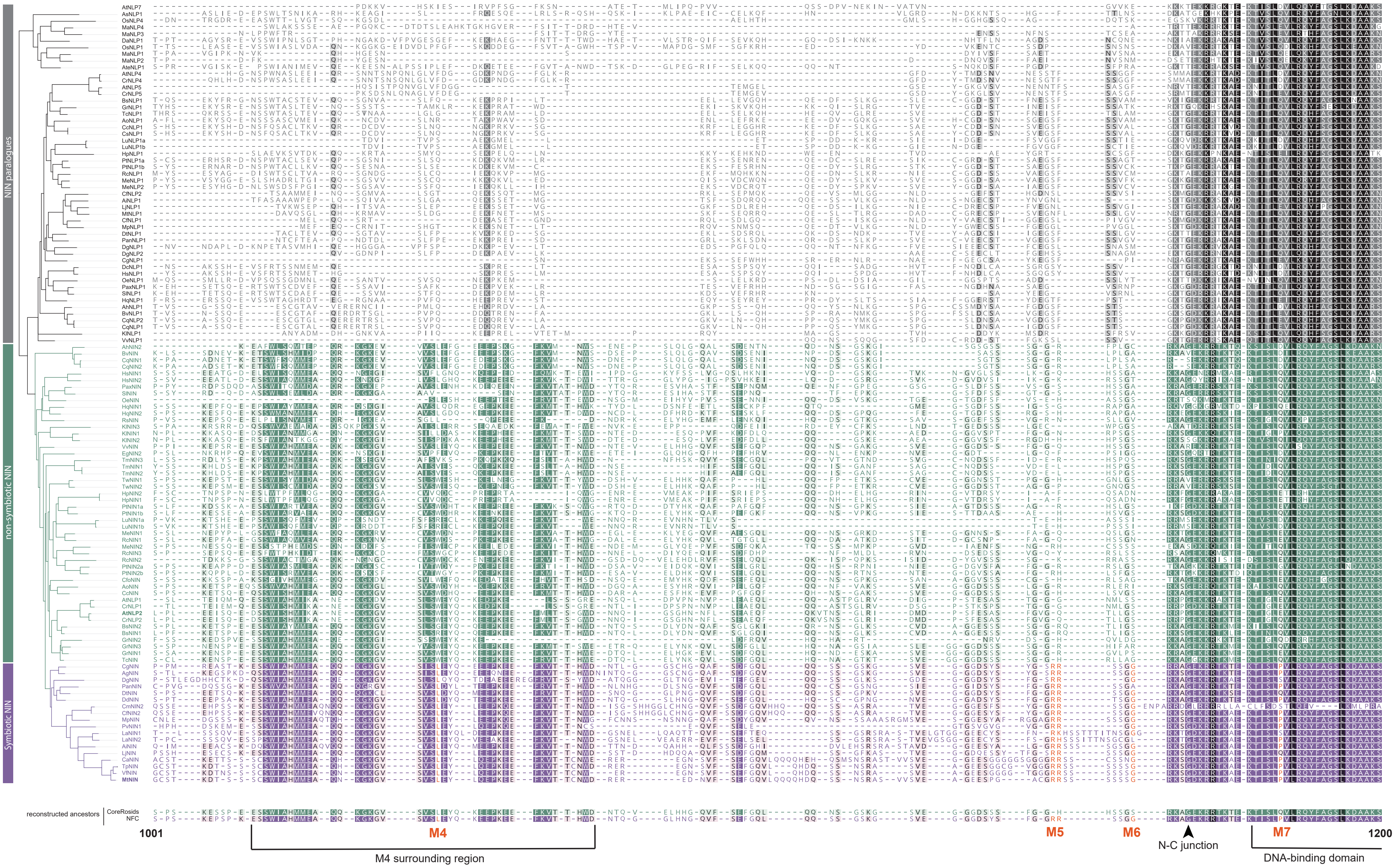
NIN paralogues
non-symbiotic NIN
Symbiotic NIN
reconstructed ancestors
CoreRosids
NFC
1001
M4
M4 surrounding region
M5
M6
N-C junction
M7
1200
DNA-binding domain

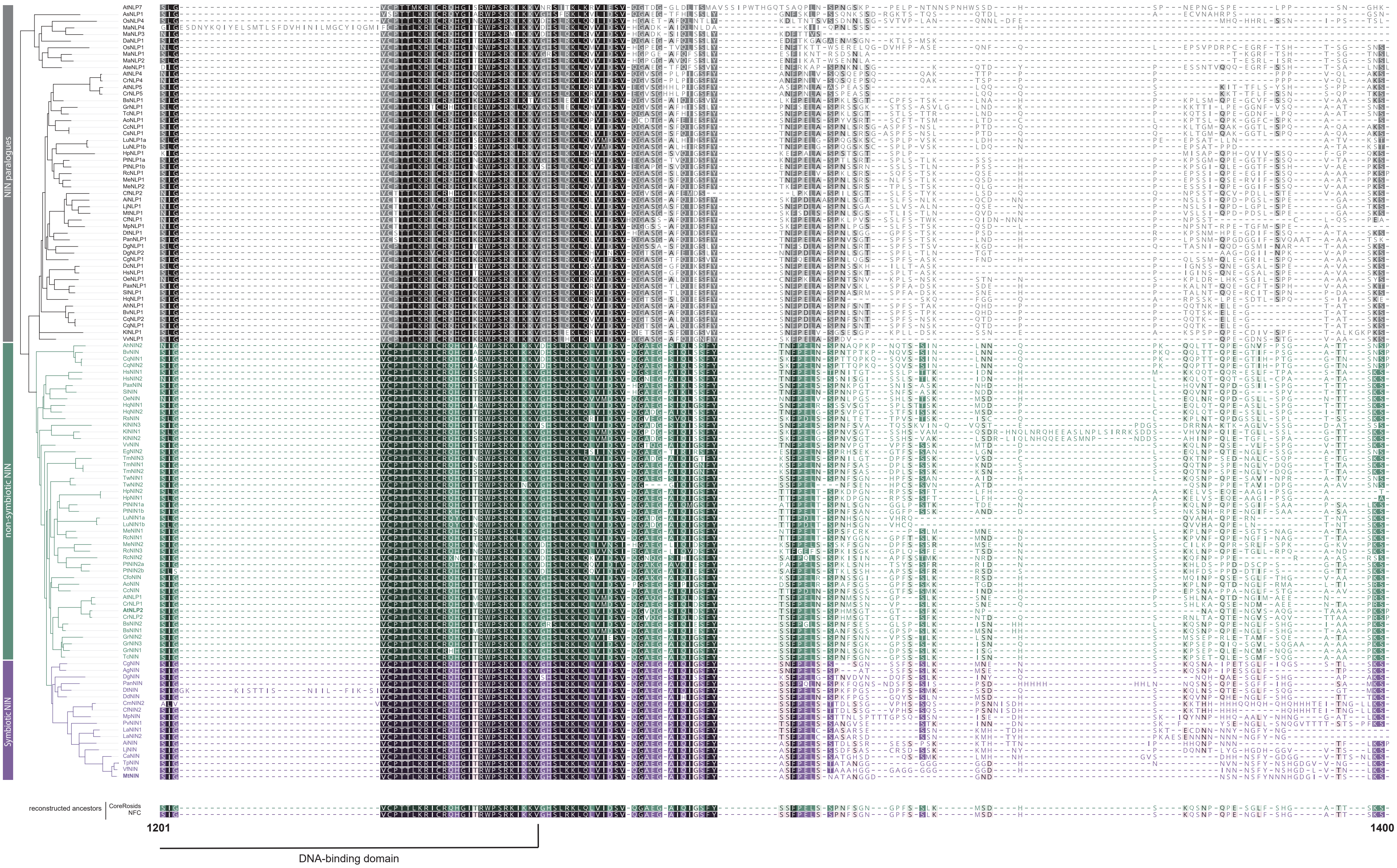
NIN paralogues
non-symbiotic NIN
Symbiotic NIN
reconstructed ancestors
CoreRosids
NFC
1201
1400
DNA-binding domain

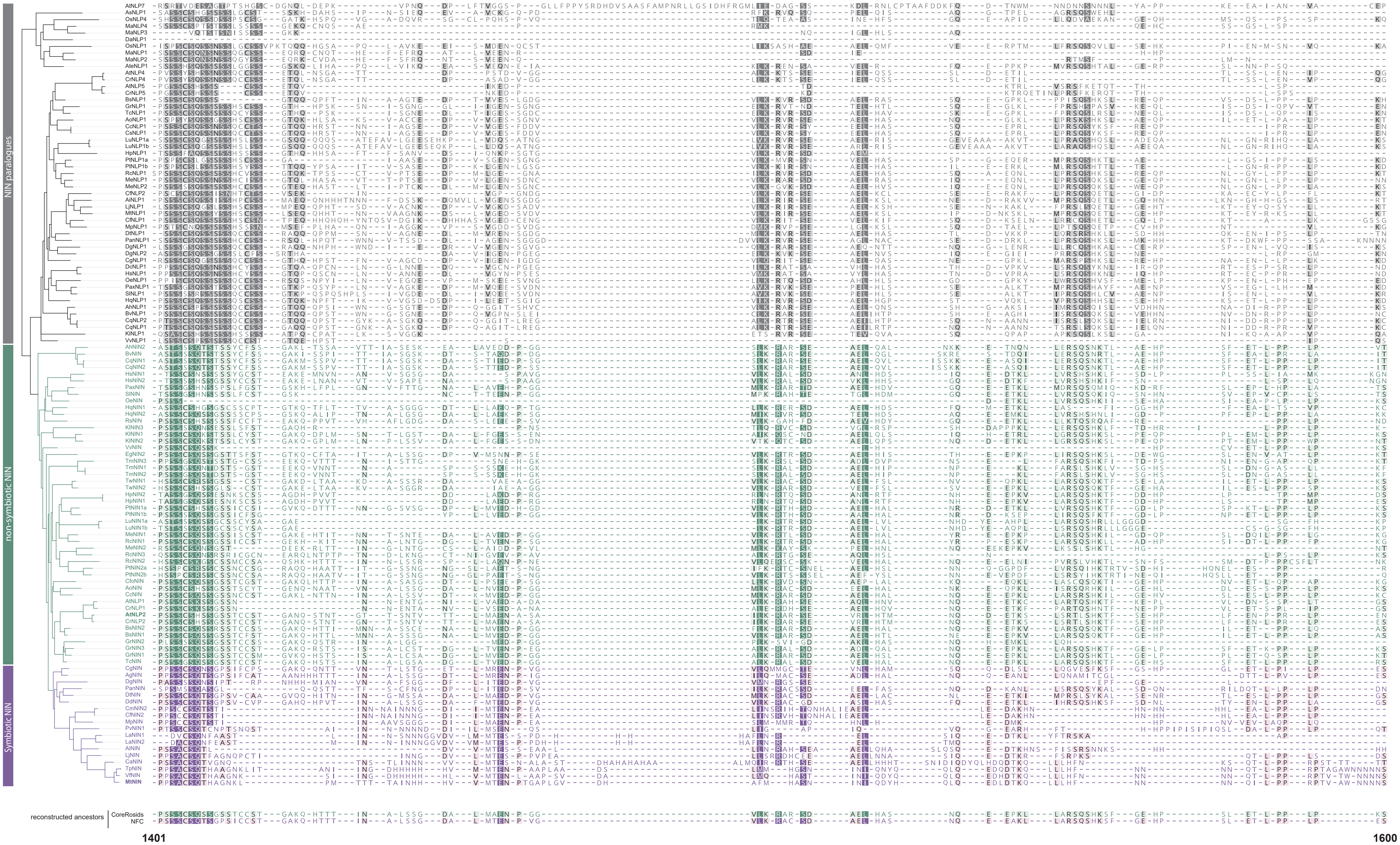
NIN paralogues
non-symbiotic NIN
Symbiotic NIN
reconstructed ancestors
CoreRosids
NFC
1401
1600

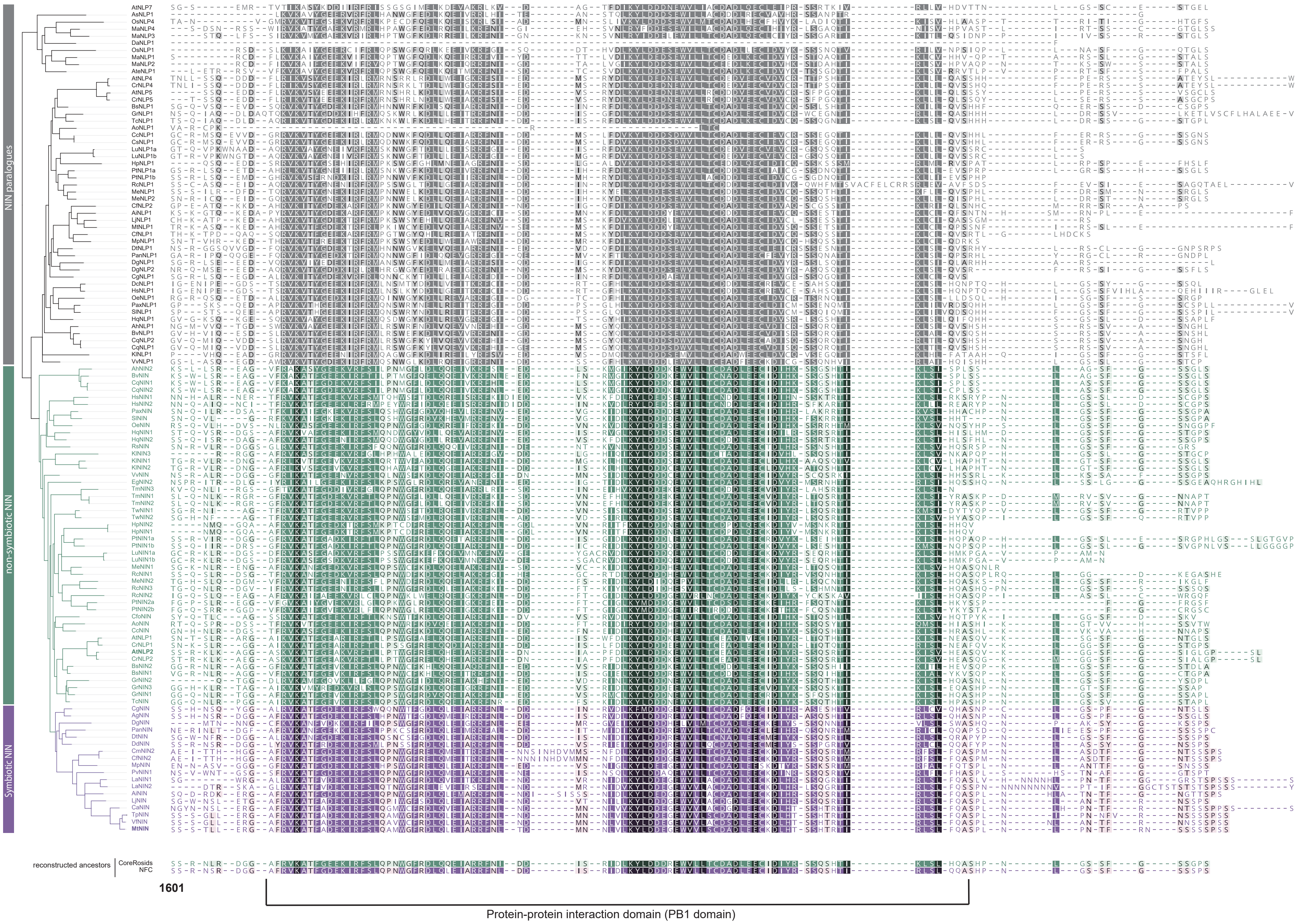
NIN paralogues
non-symbiotic NIN
Symbiotic NIN
reconstructed ancestors
CoreRosids
NFC
1601
Protein-protein interaction domain (PB1 domain)

Supplementary Fig. 7 | Alignment of NIN and NLP1 orthologs. Multiple sequence alignment of NIN and NLP1 orthologs from a range of angiosperm plant species. Purple (symbiotic NINs) and green (non-symbiotic NINs) shading indicate sequence similarity within the NIN-orthogroup; grey shading indicates sequence similarity within the non-orthologous NLPs. Seven conserved amino acid changes between symbiotic and non-symbiotic NIN orthologs (M1-7) are marked in orange. Numbers below the alignment indicate alignment position including gaps. Ac: *Aquilegia coerulea*, Ag*: Alnus glutinosa*, Ah: *Amaranthus hypochondriacus*, Ai: *Arachis ipaensis,* Ao: *Anacardium occidentale,* As: *Apostasia shenzhenica*, Ate: *Agave tequilana*, At*: Arabidopsis thaliana*, Bs: *Bretschneidera sinensis*, Bv: *Beta vulgaris*, Ca: *Cicer arietinum,* Cc: *Citrus clementina*, Cf: *Chamaecrista fasciculata*, Cfo: *Cephalotus follicularis,* Cg: *Casuarina glauca*, Cm: *Chamaecrista mimosoides*, Cq: *Chenopodium* quinoa, Cr: *Capsella rubella*, Cs: *Citrus sinensis*, Da: *Dioscorea alata*, Dc: *Daucus carota*, Dd*: Dryas drummondii*, Dg: *Datisca glomerata*, Dt: *Discaria trinervis*, Eg: *Eucalyptus grandis*, Gr: *Gossypium raimondii*, Hp: *Hypericum perforatum*, Hq: *Hydrangea quercifolia*, Hs: *Heracleum sosnowskyi*, Kl: *Kalanchoe laxiflora*, La: *Lupinus albus,* Lj: *Lotus japonicus*, Lu: *Linum* usitatissimum, Ma: *Musa acuminata*, Me: *Manihot esculenta*, Mo: *Moringa* oleifera, Mp: *Mimosa pudica*, Mt: *Medicago truncatula*, Oe: *Olea europaea*, Os: *Oryza sativa*, Pan: *Parasponia andersonii*, Pax: *Petunia axillaris*, Pt: *Populus trichocarpa*, Pv: *Phaseolus* vulgaris, Rc: *Ricinus* communis, Rs: *Rhododendron* simsii, Sl: *Solanum lycopersicum*, Tc: *Theobroma cacao*, Tm: *Tetraena* mongolica, Tp: *Trifolium* pratense, Tw: *Tripterygium* wilfordii, Vf: *Vicia* faba, Vv: *Vitis vinifera*, Zm: *Zea mays*. Vertical yellow boxes indicate the previously identified phosphorylation site[31] and predicted NIN cleavage site[11]. Arrowhead indicates the junction between N- and C-terminus that was used for the constructs in Fig. 2.

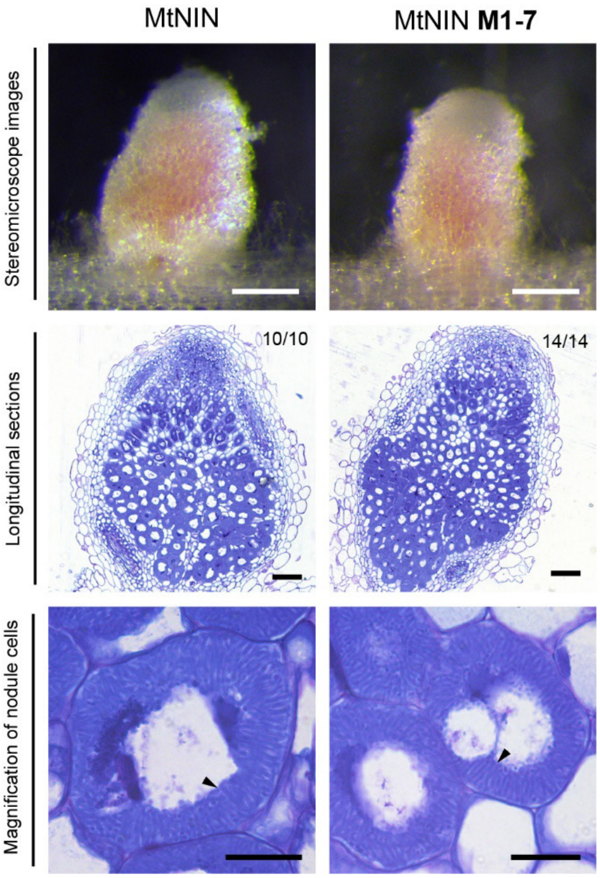

Supplementary Fig. 8 | *Mtnin-1* mutant plants complemented by MtNIN^{M1-7} form pink nodules. Nodules formed on *Mtnin-1* mutant roots complemented with MtNIN^{M1-7}, in which the seven identified residues (Fig. 4a) of MtNIN have been replaced with their AtNLP2 counterpart. Upper panels: Stereomicroscopic images showing nodules formed on roots complemented with MtNIN^{M1-7} are pink. Scale bars: 2mm. Middle panels: Longitudinal sections stained with toluidine blue. Numbers indicate nodules with released bacteria. Scale bars: 20 µm. Bottom panels: Magnification of nodule cells. Arrowheads indicate fully elongated rhizobia. Scale bars: 20 µm.

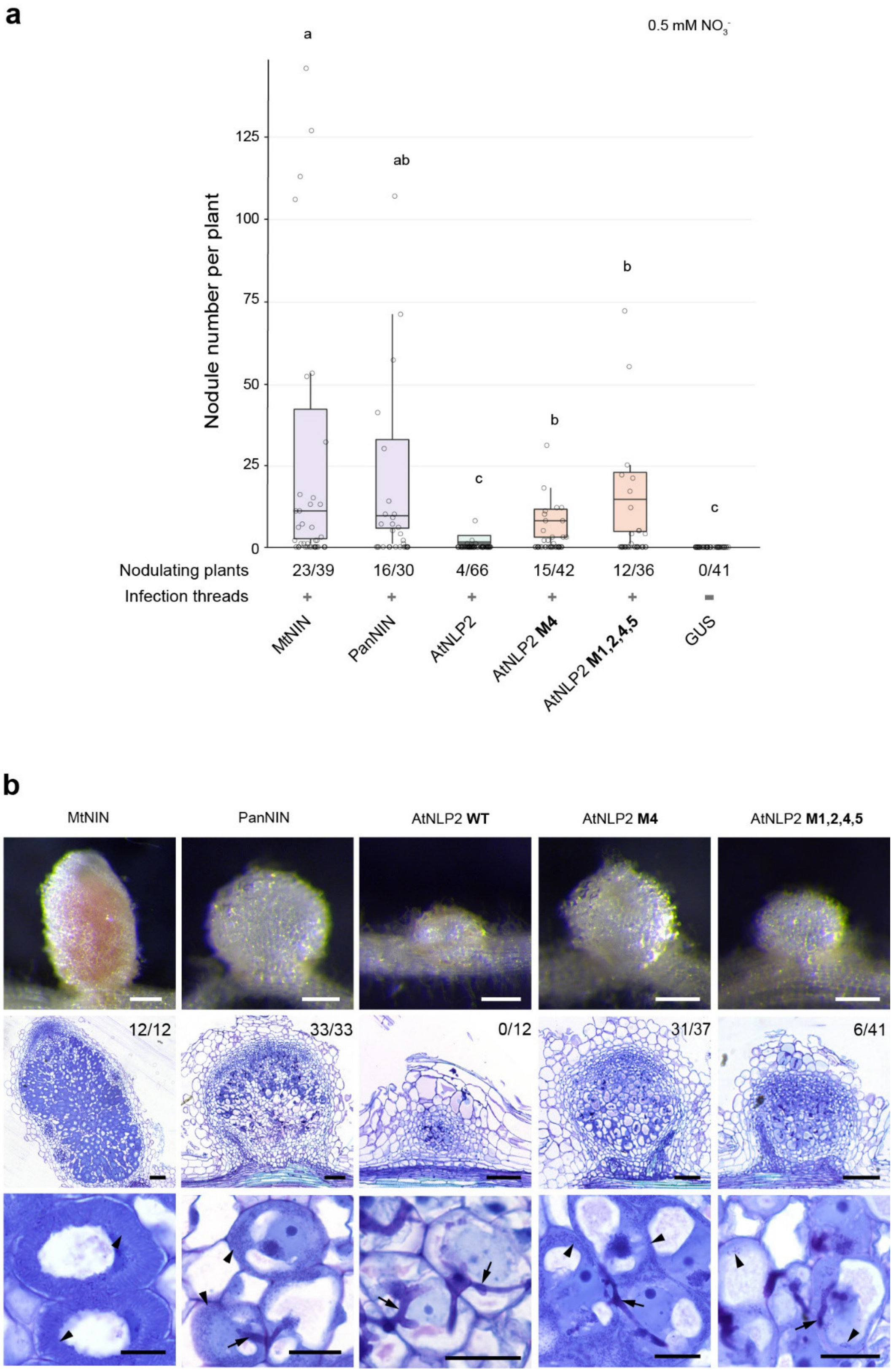

a
0.5 mM NO$_3^-$
Nodule number per plant
0
25
50
75
100
125
a
ab
b
c
b
c
Nodulating plants
23/39
16/30
4/66
15/42
12/36
0/41
Infection threads
MtNIN
PanNIN
AtNLP2
AtNLP2 M4
AtNLP2 M1,2,4,5
GUS
b
MtNIN
PanNIN
AtNLP2 WT
AtNLP2 M4
AtNLP2 M1,2,4,5
Stereomicroscope images
Longitudinal sections
12/12
33/33
0/12
31/37
6/41
Magnification of nodule cells

Supplementary Fig. 9 | AtNLP2^{M4} functions similarly to symbiotic PanNIN in nodule formation. **a**, Number of nodules formed on *Mtnin-1* mutant roots complemented with MtNIN, PanNIN, AtNLP2, AtNLP2^{M4} and AtNLP2^{M1,2,4,5}. Plants were harvested at 4 weeks post inoculation with *S. meliloti* 2011 expressing GFP. Box plots show the number of nodules per nodulated plant. Lowercase letters indicate significant differences between samples (Kruskal-Wallis and post-hoc Dunn's test, Benjamini-Yekutieli adjusted $p < 0.05$). Source data are provided as a Source Data file. All box plots show the median (centre line), interquartile range (box), and whiskers extending to the most extreme values within 1.5× the interquartile range; points represent individual observations. **b**, Images of nodules formed on *Mtnin-1* complemented with MtNIN, PanNIN, AtNLP2, AtNLP2^{M4} and AtNLP2^{M1,2,4,5}. Upper panels: Stereomicroscope images. Scale bars: 2 mm. Middle panels: Longitudinal sections stained with toluidine blue. Numbers indicate nodules with released bacteria and total nodule numbers studied. Scale bars: 100 µm. Lower panels: Magnification of nodule cells. Arrows indicate infection threads: arrowheads indicate released rhizobia. Scale bars: 20 µm.

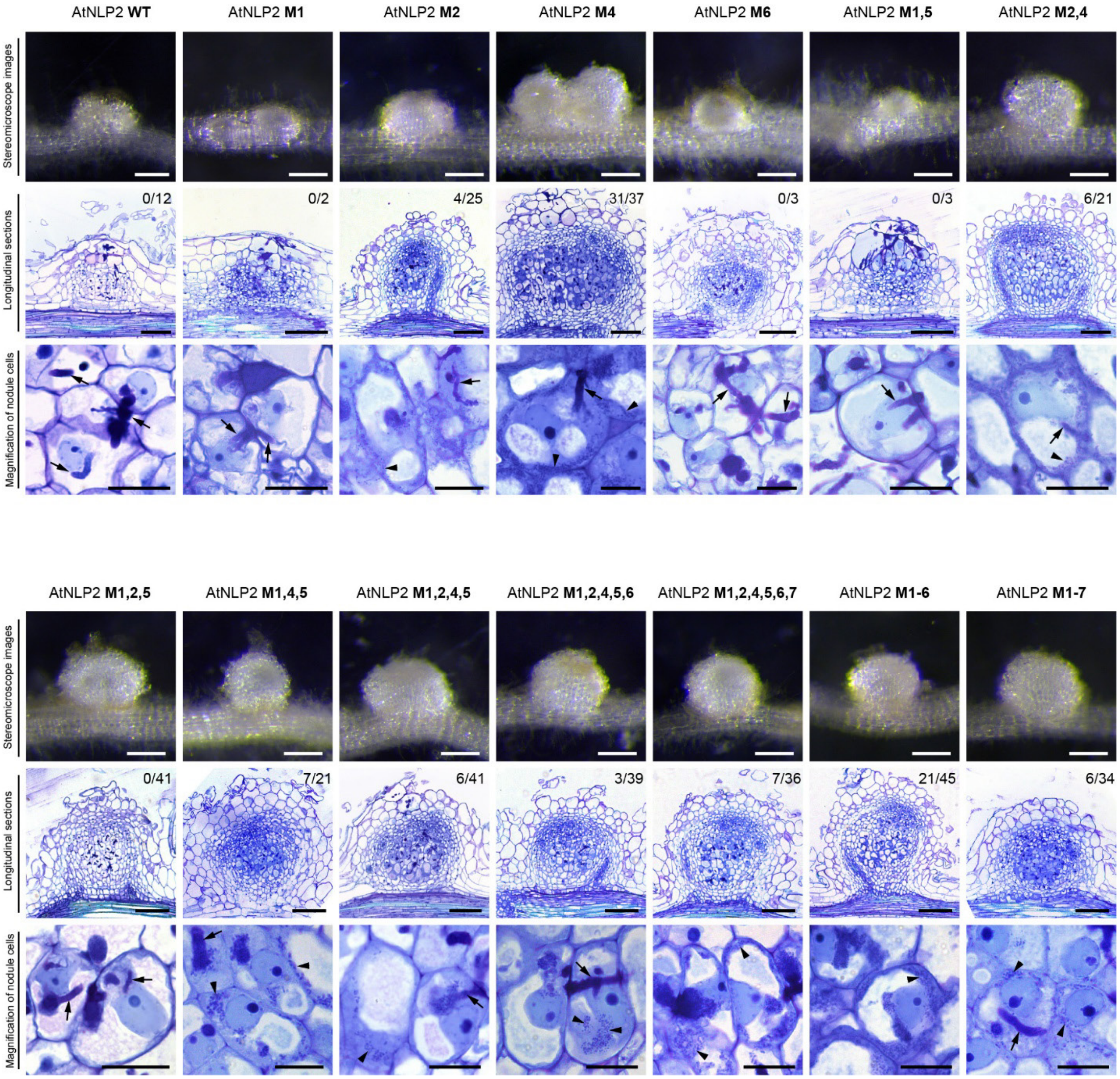

Supplementary Fig. 10 | Introducing multiple mutations into AtNLP2 did not further enhance its function in nodule formation relative to AtNLP2^{M4}. Images of nodules formed on *Mtnin-1* complemented with AtNLP2 variants, as shown in Fig. 4a,b. The combinations of multiple amino acid substitutions do not further enhance the complementation phenotype relative to the single amino acid substitution AtNLP2^{M4}. Upper panels: Stereomicroscope images. Scale bars: 2 mm. Middle panels: Longitudinal sections stained with toluidine blue. Numbers indicate nodules

with released bacteria. Scale bars: 100 µm. Lower panels: Magnification of nodule cells. Arrows indicate infection threads; arrowheads indicate released rhizobia. Scale bars: 20 µm.

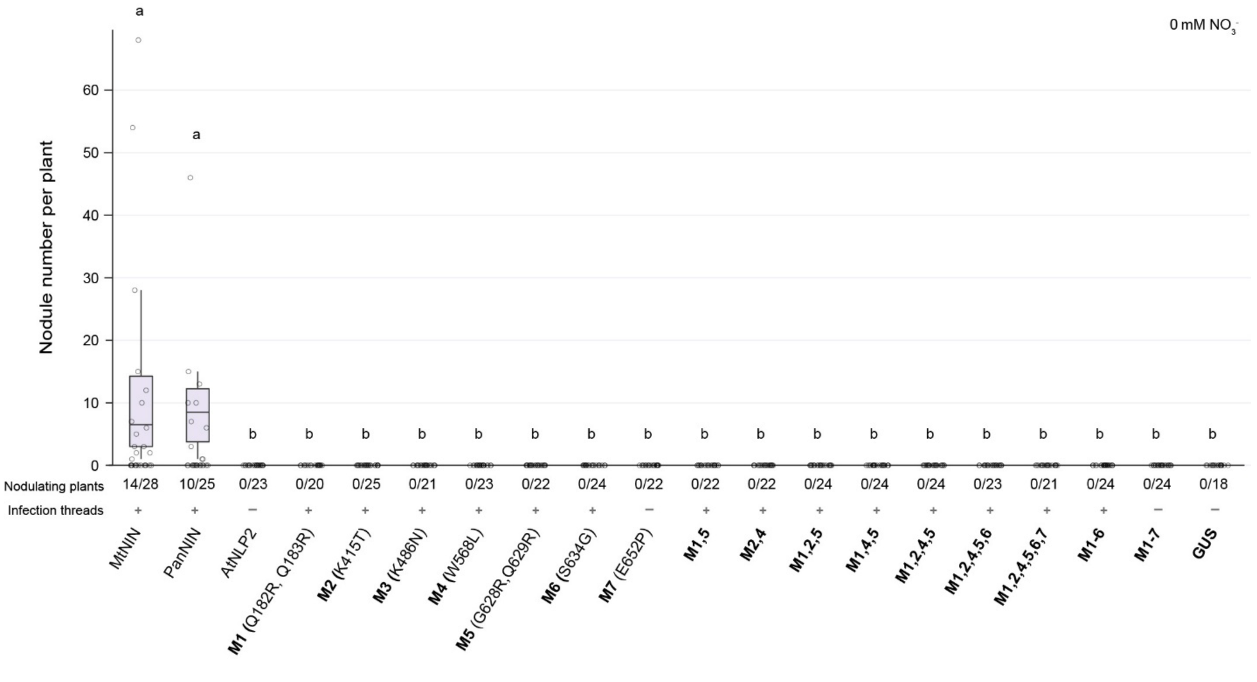

Supplementary Fig. 11 | Introducing mutations into AtNLP2 does not improve its symbiotic functionality in absence of exogenous nitrate. Number of nodules formed in absence of exogenous nitrate, on *Mtnin-1* mutant roots complemented with symbiotic NINs, wildtype AtNLP2, and AtNLP2 with different adaptations at single or multiple positions. Lowercase letters indicate significant differences between samples (Kruskal-Wallis and post-hoc Dunn's test, Benjamini-Yekutieli adjusted $p < 0.05$). Source data are provided as a Source Data file. All box plots show the median (centre line), interquartile range (box), and whiskers extending to the most extreme values within 1.5× the interquartile range; points represent individual observations.

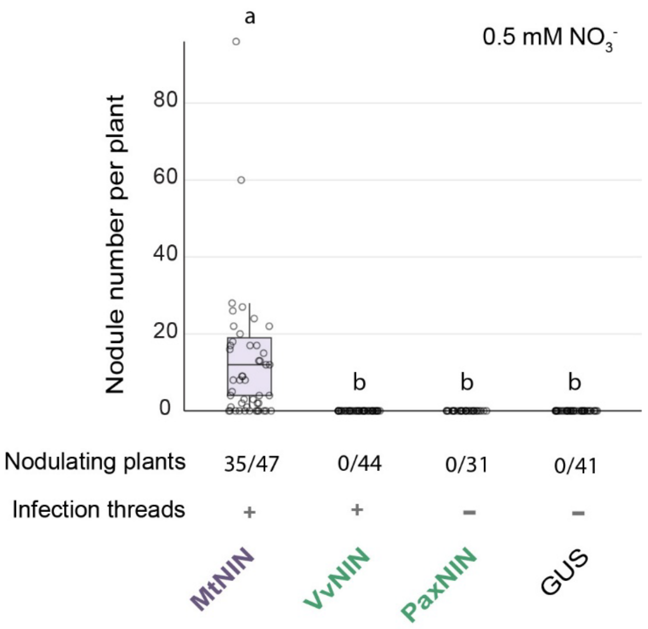

Supplementary Fig. 12 | Functionality of Petunia and Vitis NIN in nodule symbiosis. Number of nodules formed on *Mtnin-1* mutant roots complemented with NIN orthologs from *Vitis vinifera* (VvNIN) and *Petunia axillaris* (PaxNIN). Plants were harvested at 4 weeks post inoculation with Sinorhizobium meliloti 2011 expressing GFP. Box plots show the number of nodules per nodulated plant. Lowercase letters indicate significant differences between samples (Kruskal-Wallis and post-hoc Dunn's test, Benjamini-Yekutieli adjusted $p < 0.05$). Source data are provided as a Source Data file. All box plots show the median (centre line), interquartile range (box), and whiskers extending to the most extreme values within 1.5× the interquartile range; points represent individual observations.

Supplementary Table 1
Primers used in this study

**Primer name**	**Sequence 5' -> 3'**
MtNF-YA1-qPCR-F	TATGGAGGAGACTCTTGTGG
MtNF-YA1-qPCR-R	GGTTGCTTGATGATTTGGTG
MtCEP7-qPCR-F	CAAGCCAGGCCAATTAAACC
MtCEP7-qPCR-R	TCGGCCGAAAAGAGTTAGTG
MtCLE13-qPCR-F	TCAACTTTGCAGGCTCGTAG
MtCLE13-qPCR-R	TGTAGAAGGCTTCGGCTGTC
MtLBD16-qPCR-F	AGCTCGTATCAGAGACCCT
MtLBD16-qPCR-R	TGCAAGCATGCTACCTGTTGTTG
MtNF-YB16-qPCR-F	TGATGAAAACGGAGGCATAA
MtNF-YB16-qPCR-R	AGTGTACCCAAAGCCCAACA
MtACTIN2-qPCR-F	TGGCATCACTCAGTACCTTTCAACAG
MtACTIN2-qPCR-R	ACCCAAAGCATCAAATAATAAGTCAACC
EMSA probe MtNF-YA1 sense	AGTAGACCCTTTATAAGTTCCAAGAATAA
EMSA probe MtNF-YA1 antisense	GGAGTTATTCTTGGAACTTATAAAGGGTC
EMSA probe MtNF-YB16 sense	GGAGTGCATCTTTAGACCTTTCCAAGGGA
EMSA probe MtNF-YB16 antisense	AGTATCCCTTGGAAAGGTCTAAAGATGCA
EMSA probe MtCEP7 sense	GGAGTTAATTTTTAAGTACACCAAAGGACA
EMSA probe MtCEP7 antisense	AGTATGTCCTTTGGTGTACTTAAAAATTAA
EMSA probe MtCLE13 sense	GGAGTGACCCTTAAGTCTTGGAGGGGA
EMSA probe MtCLE13 antisense	AGTATCCCCTCCAAGACTTAAGGGTCA
EMSA probe MtLBD16 sense	AGTACTGGCCCTTGAAGCATCAAAGAGC
EMSA probe MtLBD16 antisense	GGAGGCTCTTTGATGCTTCAAGGGCCAG
EMSA-IR700-F	CATTAATCACTCTGTGGTCTCAGG
EMSA-IR700-R	CTGAAGAGCCACTTCGTGGT
