# Supplementary material for: Ancestral functionality and symbiotic refinement of NIN in root nodule symbiosis
Source: Nat Commun. 2026 Apr 6;17:4907. doi: 10.1038/s41467-026-71330-1 (PMC13230612; doi:10.1038/s41467-026-71330-1)
Supplement: Supplementary file 2 — Description of Additional Supplementary Files [file 41467_2026_71330_MOESM2_ESM.pdf]

Description of Additional Supplementary Files

File Name: Supplementary Data 1

Description: Comparison of codon usage between different NIN orthologs and the Medicago truncatula genome.

File Name: Supplementary Data 2

Description: Ancestral sequence reconstruction and analysis of amino acid conservation.

File Name: Supplementary Data 3

Description: Constructs used in this study.

File Name: Supplementary Data 4

Description: Protein sequences used for analysis.
